# Reproductive inequalities in the acanthocephalan *Corynosoma cetaceum*: looking beyond ‘crowding’ effects

**DOI:** 10.1186/s13071-018-2723-x

**Published:** 2018-03-21

**Authors:** Francisco Javier Aznar, Jesús Servando Hernández-Orts, Gabriela Vélez-Rubio, Luis M. Fernández, Nadia T. Muriel, Juan Antonio Raga

**Affiliations:** 1 Instituto Cavanilles de Biodiversidad y Biología Evolutiva, Parque Científico, Universidad de Valencia, Paterna, Valencia, España; 2 Centro de Investigación Aplicada y Transferencia Tecnológica en Recursos Marinos Almirante Storni (CIMAS - CCT CONICET - CENPAT), San Antonio Oeste, Río Negro Argentina; 3 Karumbe NGO, Av. Rivera 3245, CP 11400 Montevideo, Uruguay; 4 Centro Universitario Regional del Este (CURE), Universidad de la República, Sede Rocha, Intersección Ruta 9 y Ruta 15, Rocha, Uruguay; 5 Ceiba Foundation for Tropical Conservation, 301 S. Bedford Street, Suite 7A, Madison, WI 53703 USA; 6 Padre Lozano 329, Barrio Alto Alberdi, Córdoba, Argentina

**Keywords:** Acanthocephala, Polymorphidae, *Corynosoma*, Inequalities, Fecundity, Competition, Body size

## Abstract

**Background:**

At present, much research effort has been devoted to investigate overall (“average”) responses of parasite populations to specific factors, e.g. density-dependence in fecundity or mortality. However, studies on parasite populations usually pay little attention to individual variation (“inequality”) in reproductive success. A previous study on the acanthocephalan *Corynosoma cetaceum* in franciscana dolphins, *Pontoporia blainvillei*, revealed no overall intensity-dependent, or microhabitat effects, on mass and fecundity of worms. In this study, we investigated whether the same factors could influence mass inequalities for this species of acanthocephalan.

**Methods:**

A total of 10,138 specimens of *C. cetaceum* were collected from 10 franciscanas accidentally caught in Buenos Aires Province between 1988 and 1990. To investigate mass inequalities, all the specimens were sexed, and females were classified according to their developmental stage and weighed. Additionally, the relationship between biomass and fecundity (estimated as the number of acanthors) was investigated for some females. Inequalities in fecundity and biomass were assessed using standard methods, i.e. the Lorenz curve and the Gini coefficient (G).

**Results:**

We found a modest, but highly significant linear relationship between mass and fecundity. The G was very low (0.314) compared with that from other helminth species. G values were significantly lower in gravid females, which presumably exhibit a slow rate of growth. Also, G values significantly increased with total intensity, but only for gravid females, and the effect was more predictable considering only the intensity of gravid females.

**Conclusions:**

Apparently, high intensity infections increase inequality of reproducing females without producing “crowding” effects. Although several processes could generate this pattern, gravid females, at higher intensities, expanded their distribution and occupied gut chambers with contrasting environmental conditions, which might result in greater variability in body size. The observed inequalities are not expected to strongly influence the population genetics of *C. cetaceum*, but they reveal subtle individual effects beyond an overall population impact.

**Electronic supplementary material:**

The online version of this article (10.1186/s13071-018-2723-x) contains supplementary material, which is available to authorized users.

## Background

A major goal of studies on population dynamics of parasites is to identify the factors that drive changes of population size and genetics over ecological and microevolutionary timeframes [1–3]. Much research effort has been devoted to investigate overall (“average”) responses of parasite populations to specific factors, e.g. density-dependence in fecundity or mortality [4, 5]. Little attention has been paid, however, to the influence of individual variation (“inequality”) in reproductive success on population dynamics [6]. High inequality reduces the effective population size, which may result in faster response to selection or greater genetic drift of parasite populations [7]. For instance, in a population of the cestode *Triaenophorus crassus* infecting pikes, *Esox lucius*, just about 10% of worms accounted for 85% of all parasite fecundity, and 55% of worm eggs were produced in just 10% of pike [8].

To date, the available studies reporting measurement of reproductive inequality in parasites have dealt with experimental infections of cestodes and acanthocephalans [6], or natural populations of digeneans [9], cestodes [8], nematodes [10–13] and nematomorphs [7]. Using different proxies of reproductive success, such as body mass or length, number of ovarian balls or eggs or lifetime fecundity, these studies revealed substantial variation in the level of inequality among parasite taxa, particularly between helminths with indeterminate *vs* determinate growth [6, 7]. Also, some studies have identified factors that significantly influence inequality levels, including the mode of infection, i.e. trickle *vs* clumped [12, 13], the level of critical nutrients [6], or the number of parasites in the infrapopulations, i.e. intensity-dependent effects [6].

One of the most interesting findings of these studies is that the same factors can lead to decoupled responses at individual (i.e. inequalities) and population (“average”) levels. For instance, Keymer et al. [14] found that worm density exerted a greater effect than host nutrition on the mean worm size of the cestode *Hymenolepis diminuta* in rats, whereas Dobson [6], using the same dataset, found greater effects of host nutrition than worm density when focusing on size inequalities. Likewise, Keymer et al. [15] investigated the combined effect of cystacanth dose and the concentration of a critical nutrient (fructose) on the average length of the acanthocephalan *Moniliformis moniliformis* infecting rats. These authors discovered significant ‘crowding’ effects, but failed to detect a significant interaction between cystacanth dose and nutrient concentration. However, Dobson [6] did report strong interactions between these factors with regard to reproductive inequalities. In particular, low fructose concentrations disproportionately increased inequalities at higher parasite doses.

In this study, we report, for the first time, on reproductive inequalities in a natural population of an acanthocephalan, namely, *Corynosoma cetaceum* in franciscana dolphins, *Pontoporia blainvillei,* based on measurements of body mass. This system is interesting for two reasons. First, the parasite is able to mature and reproduce in several microhabitats with contrasting physical and chemical conditions [16]; this allowed us to explore whether microhabitat variability may impact reproductive inequalities, an issue that has never been explored for parasites. In particular, individuals of *C. cetaceum* are found in three gut chambers: (i) the main stomach (MS), a pear-shaped chamber where entire prey are stored and converted to semi-fluid chyme through mechanical and chemical digestion at low pH; (ii) the pyloric stomach (PS), a J-shaped, elongated tubular chamber where the chyme coming from the MS is neutralized by mucous secretions before entering the duodenum through the pyloric sphincter; and (iii) the duodenal ampulla (DA), which is a funnel-shape expansion of the proximal duodenum that receives hepatic and pancreatic juices for further digestion of food [17–19].

A further interesting feature of infection of *C. cetaceum* in franciscanas is that no significant effects of parasite intensity, or microhabitat, have been detected on the mean mass or fecundity of gravid females [16]. However, in this study we will show that mass inequalities of *C. cetaceum* are significantly associated with both factors. This finding stresses the importance of considering parameters other than mean reproductive effort, i.e. inequalities, to better understand the population dynamics of parasites.

## Methods

### Data collection

Individuals of *C. cetaceum* were collected from franciscanas accidentally caught in shark fisheries from Buenos Aires Province, Argentina in 1988–1990 (see Aznar et al. [16] for details). Worms were collected from the MS, the PS, and the DA, and fixed and preserved in 70% ethanol. The approximate relative area of each chamber (in percentage), calculated based on figure 1 from Aznar et al. [16] is 69.1% for the MS, 25.3% for the PS (including the connecting channel) and 5.6% for the DA.

In 10 franciscanas used in the study by Aznar et al. [16], all individuals of *C. cetaceum* were counted and sexed. Females were cleared in lactophenol and classified under a stereomicroscope as stage 1 (ovarian balls only), stage 2 (containing a mixture of ovarian balls and developing acanthors) and stage 3 (containing a mixture of developing acanthors and fully developed acanthors) [16, 20]. After classification, lactophenol was removed from females by washing them in 70% ethanol, and they were returned to vials with this preservative. For weighing, females were left overnight in refrigerated tap water and, prior to weighing, each specimen was briefly put on dry paper to remove the excess of water and was individually weighed with a precision scale to the nearest 0.1 mg.

To investigate the relationship between mass and fecundity (estimated as number of acanthors) we selected, in 15 additional franciscanas from the study by Aznar et al. [16], 5 females of stage 3 from each chamber (*n* per host = 15, total *n* = 225 females) that were preserved in 70% ethanol. Each specimen was weighed as described above and their contents were washed into a beaker with 10 ml of distilled water. The contents were agitated with a magnetic stirrer for 5 min, and ten samples of 10 μm were taken and the number of acanthors counted. The average number of acanthors from these samples was extrapolated to the total volume.

### Statistical analyses

General linear mixed models with type I sum of squares were used to investigate the relationship between number of eggs (dependent variable) and mass (fixed covariate). ‘Chamber’ (fixed factor, 3 levels) and the interaction ‘chamber*mass’ were included as additional predictors, and ‘individual host’ as a random factor.

Inequalities in fecundity and biomass were assessed using standard methods, i.e. the Lorenz curve and the Gini coefficient (e.g. [6–8, 11, 12]). Lorenz curves were obtained by ordering worms in specific samples (e.g. stage-3 females) from the smallest to the largest and plotting the cumulative biomass against the cumulative number of ordered worms; a more pronounced concavity would imply a higher inequality [6]. The Gini coefficient (hereafter referred to as G) was used to quantify this inequality. G measures the ratio between the area defined by the diagonal (i.e. the situation in which all worms have the same weight) and the observed Lorenz curve over the triangular area under the diagonal [21]. It can take values from 0 (all worms have the same weight) to 1 (the theoretical maximum in an infinite population in which all worms but one have a value of 0).

G is a summary parameter the value of which depends on the specific subset of individuals used to calculate it. It was not possible to investigate the conjoint effect of stage and gut chamber on G because subdivision of females into 9 categories would excessively reduce the sample size to calculate G in many subsets (*n* < 10 in nearly half of subsets, see Additional file 1: Table S1). Instead, we adopted a simpler exploratory approach. To investigate stage effects on inequality we calculated G for each stage per host, regardless of chamber, and compared them by repeated measures ANOVA. To investigate the effect of intensity and microhabitat on G values for each stage, zero-order and partial Pearson correlations were used. Microhabitat effects were assessed based on two parameters, i.e. mean worm position and its standard deviation (SD) [22]. These parameters were calculated as follows: each worm scored as the chamber where it occurred (1 for MS, 2 for PS and 3 for DA). Then, for each host individual, an average position (i.e. the “mean” worm) was calculated, and the associated SD was taken as an index of microhabitat variability. The SD was preferred to an index of niche breadth because the SD conserves information on chamber order. For instance, the niche breadth index would be the same for two hosts, one harboring *x* worms in MS and *y* in PS, and the other harboring *x* in MS and *y* in the AD. However, SD would be higher in the second case, which makes more ecological sense.

Lorenz curves were represented graphically using the package ‘*ineq*’ for R [23]. G values (corrected for sample size), were calculated with package ‘*reldist*’ for R [24], with 95% confidence intervals (CI) being set by simple bootstrap based on 20,000 resamples using the ‘*boot*’ package [25]. The remaining analyses were carried out with the package SPSS v22.

## Results

### Population structure

The intensity of *C. cetaceum* in the 10 franciscanas ranged between 99–2692 individuals (Additional file 2: Table S2), with a total of 10,138 individuals. Worms were found attached on the aboral part of the MS, and covering the wall of the PS and the DA. On average, about three-quarters of worms (mean per dolphin ± SD: 75.0 ± 13.0%) were found in the PS; the relative proportions of worms in the MS and the DA were similar (11.7 ± 10.0% *vs* 13.3 ± 5.5%) (Additional file 2: Table S2). This pattern was highly repeatable from host to host (Kendall test, *W* = 0.76, *df* = 2, *P* = 0.001).

The overall sex ratio was female-biased, but the MS was relatively enriched with males (mean sex ratio ± SD: 51.4 ± 7.8%) compared with the PS (42.3 ± 9.1%) and the DA (45.3 ± 11.2%) (Additional file 2: Table S2). Likewise, there were comparatively more stage-1 females in the MS (mean percent per host ± SD: 56.0 ± 28.3%) than in the PS and AD, whose relative numbers were similar (30.2 ± 18.0% and 30.2 ± 23.4%, respectively) (Additional file 1: Table S1). The proportions of stage-2 and stage-3 females were also comparable between PS (17.0 ± 8.4 and 52.8 ± 23.2, respectively) and AD (18.9 ± 18.9 and 50.8 ± 33.3, respectively). At infrapopulation level, stage-3 females were the most numerous (mean percent per host ± SD: 50.3± 24.3%) followed by stage-1 (33.0 ± 19.6%) and stage-2 (16.7 ± 9.1%) females (Additional file 1: Table S1). This pattern was moderately repeatable from host to host (Kendall test, *W* = 0.31, *df* = 2, *P* = 0.045).

### Relationship between biomass and fecundity

**Fig. 1:**
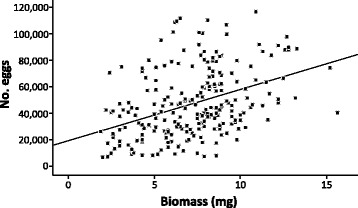
Regression line of number of eggs on mass for stage-3 females of the acanthocephalan *Corynosoma cetaceum*. Worms were collected from the main stomach, pyloric stomach, and duodenal ampulla from 15 franciscana dolphins, *Pontoporia blainvillei*

**Fig. 2:**
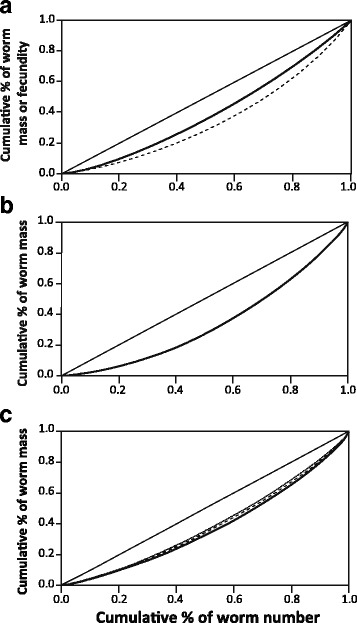
Lorenz curves for females of *Corynosoma cetaceum* infecting franciscana dolphins, *Pontoporia blainvillei*. Cumulative worm mass, or fecundity, are plotted against cumulative percent of worm number. Diagonals represent equality of all individuals. **a** Curve for mass (solid line) and fecundity (broken line) for a subsample of 225 individuals collected from 15 hosts. **b** Curve for mass for the total female sample (*n* = 5368) collected from 10 hosts. **c** Curve for mass for each of three development stages of females: 1 (solid line), 2 (broken line) and 3 (dotted line), based on the same sample

Mass of stage-3 females from the subsample of worms (*n* = 225) from 15 franciscanas ranged between 1.9–15.2 mg (mean ± SD: 7.21 ± 2.64), and fecundity 6,800–116,600 eggs (47,164 ± 25,532). There was a significant linear relationship between mass and fecundity (*F*_(1, 223)_ = 42.37, *P* < 0.0001) (Fig. 1), although the variance explained was low (*R*^2^ = 0.160). No significant effects of chamber, or the interaction chamber*mass, were significant (*P* > 0.05). The Lorenz curve for mass of *C. cetaceum* in this worm sample showed only a slight degree of concavity (Fig. 2a), with a low Gini coefficient (G = 0.206, 95% CI: 0.194–0.218). The concavity of the Lorenz curve was more pronounced for fecundity (Fig. 2a), and the Gini coefficient was correspondingly higher (G = 0.303, 95% CI: 0.289–0.317).

### Inequalities in biomass

Mean mass (SD) of females of *C. cetaceum* in the overall sample was 5.63 ± 3.19 mg, with a range between 0.9–24.0 mg (*n* = 5368). The increase in mass from stage-1 to stage-3 females was roughly linear in both the mean values for total sample (stage 1: 2.86 ± 1.42; stage 2: 4.92 ± 2.06; stage 3: 7.42 ± 2.90) and the average of mean values per host (stage 1: 2.95 ± 0.84; stage 2: 4.72 ± 1.41; stage 3: 7.12 ± 1.88).

In the total sample, the Lorenz curve for biomass was slightly concave (Fig. 2b), with an associated G of 0.314 (95% CI: 0.310–0.317). Inequalities did not differ broadly among developmental stages but there was a significant decrease of G from stage 1 to 3: stage 1 (G = 0.255, 95% CI: 0.249–0.261, *n* = 1739), stage 2 (G = 0.227, 95% CI: 0.219–0.235, *n* = 676), and stage 3 (G = 0.212, 95% CI: 0.208–0.216, *n* = 2953) (Fig. 2c). This pattern was confirmed using G values per host for each stage (repeated measures ANOVA, linear polynomial contrast, *F*_(1,19)_ = 5.534, one-tailed *P* = 0.021). The value of G for gravid females (i.e. stages 2–3 pooled), was 0.216 (95% CI: 0.201–0.231).

**Fig. 3:**
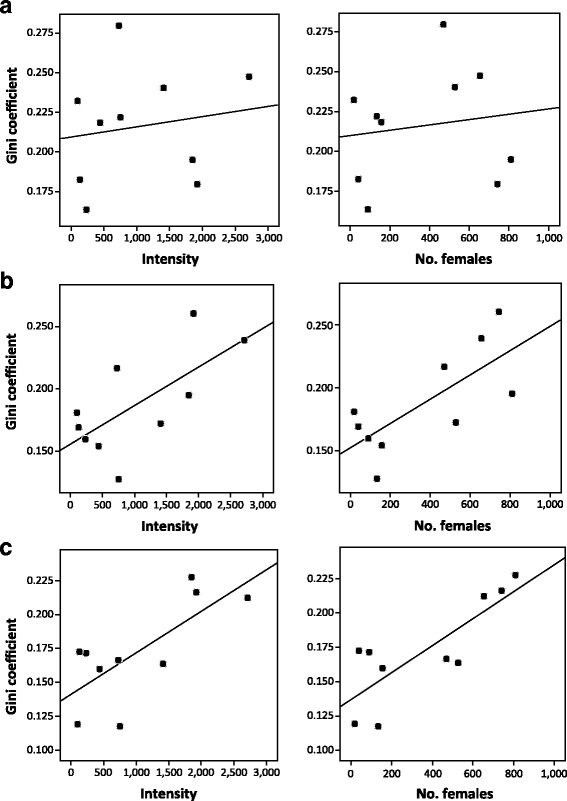
Regression lines of the Gini coefficient for three development stages of females of *Corynosoma cetaceum* on total number of worms (intensity) or total number of gravid females (number of females). **a** Stage 1. **b** Stage 2. **c** Stage 3. Data come from infrapopulations of 10 franciscana dolphins, *Pontoporia blainvillei*

The matrix of Pearson correlations between G of each stage and 3 predictors (intensity, mean worm position, and SD of mean worm) is shown in Table 1. None of the predictors had a significant effect on G for stage-1 females, but intensity showed a significant positive relationship for both stage-2 and stage-3 females (Fig. 3). In addition, G significantly increased with SD of the mean worm (Table 1). The observation that only gravid females were affected by intensity and habitat variability led us to hypothesize that they could experience intensity-dependent effects more intensely. Thus, we repeated the analysis considering the number of gravid females as an alternative predictor of intensity. In this case, the correlation between G and number of gravid females remained non-significant for stage-1 females, but the coefficient of correlation clearly increased in both stage-2 and stage-3 females (Table 1, Fig. 3).

**Table 1:** Matrix of Pearson correlation coefficients for the Gini coefficient (G), the intensity (or the number of gravid females), the mean worm position, and the standard deviation (SD) of the mean worm, per host, for females of three developmental stages of the acanthocephalan *Corynosoma cetaceum* in ten franciscana dolphins, *Pontoporia blainvillei*. Correlations in bold remain significant after the sequential Bonferroni correction

	Intensity (No. of females)	Mean worm	SD
Stage 1			
G	0.161 (0.144)	0.520	-0.146
Intensity (No. of females)	–	0.272 (0.203)	-0.579 (-0.435)
Mean worm	–	–	-0.036
Stage 2			
G	**0.681** ^*^ **(0.733)** ^**^	0.229	0.141
Intensity (No. of females)	–	0.107 (-0.029)	0.066
Mean worm	–	–	0.311
Stage 3			
G	**0.733** ^**^ **(0.810)** ^****^	0.244	**0.756** ^**^
Intensity (No. of females)	–	0.527	**0.807** ^***^ **(0.686)** ^*^
Mean worm	–	–	0.120

*P < 0.025

**P < 0.01

***P < 0.005

****P < 0.0025

The intensity (or the number of gravid females) did not affect the position of the mean worm for any stage but, in stage-3 females, the SD of the mean worm was higher at increasing intensities (or numbers of gravid females) (Table 1). This suggests that density-dependence could contribute to increase microhabitat variability. Thus, partial correlations were used to explore the separate effects of population size and microhabitat variability on G (Table 2). Although the power of the tests was low (*df* = 7), we found a significant correlation between G and intensity (stage 2), and G and number of gravid females (stages 2 and 3), after controlling for the SD of the mean worm (Table 2). However, the partial correlations between G and SD controlling for intensity or number of gravid females were lower and non-significant (Table 2).

**Table 2:** Partial Pearson correlation coefficients (with nominal *P*-values in parentheses) between the Gini coefficient, and intensity or number of gravid females [controlled for the standard deviation (SD) of mean worm], and between the Gini coefficient and SD (controlled for intensity, or the number of gravid females) for individuals of the acanthocephalan *Corynosoma cetaceum* collected in ten franciscana dolphins, *Pontoporia blainvillei*. Correlations in bold remain significant after the sequential Bonferroni correction

Controlling variable	Predictor	Stage 1	Stage 2	Stage 3
Intensity	SD	-0.066 (0.433)	0.131 (0.369)	0.409 (0.137)
SD	Intensity	0.095 (0.404)	0.680 (**0.022**)	0.319 (0.202)
No. of females	SD	-0.094 (0.405)	-0.108 (0.391)	0.469 (0.101)
SD	No. of females	0.090 (0.409)	0.730 (**0.013**)	0.611 (**0.040**)

## Discussion

To date, the few studies that have investigated reproductive inequalities in parasites have dealt with different proxies of reproductive output, including worm length, worm mass, number of ovarian balls (in acanthocephalans) or fecundity [6–12]. Worm size is assumed to be positively related with fecundity [11–13] and, indeed, such relationship was found to be statistically significant in the three studies on inequality in which it was analyzed. However, the percent variance in fecundity that was accounted for by worm size was highly variable, with a low value of *R*^*2*^ (0.285) for the nematode *Raphidascaris acus* infecting pikes [10], medium (0.504) for the cestode *Triaenophorus crassus* from pikes [8], and high (0.881) for the nematomorph *Paragordius varius* from crickets, *Grillus firmus* [7]. In the present study, just a modest portion of variability in fecundity of *C. cetaceum* was accounted for by worm mass (*R*^2^ = 0.160). It should be noted, however, that the fecundity estimates vary in accuracy, particularly when compared with mass measurements. In the case of *T. crassus* and *P. varius*, the eggs counted represented the whole egg output of each individual. However, in *R. acus* and *C. cetaceum*, only a snapshot of egg production (i.e. the number of eggs *in utero* or body cavity) was obtained, and thus a weaker relationship is perhaps not surprising in this case.

In addition, values of G are not necessarily comparable among studies because measurement error could vary for different proxies of reproductive output. In the only two studies in which G was calculated for both worm fecundity and size, the former was consistently higher: 0.90 (fecundity) *vs* 0.61 (mass) for *T. crassus* [8] and 0.73 (fecundity) *vs* 0.60 (mass) for *R. acus* [10]. Our results for *C. cetaceum* would agree with this pattern because, in a random subsample of worms, G was significantly higher for fecundity than for mass (0.303 *vs* 0.206). However, one could wonder to what extent measurement error differs in estimates of fecundity *vs* mass, and to what extent this difference may influence the corresponding G values. We feel that such influence is most likely negligible because measurement error, regardless of magnitude, is expected to be randomly distributed among individuals. To explore this, we re-weighed 20 gravid females 10 times, and calculated the coefficient of variation (CV) (%) of mean weight per worm, which ranged between 0.77–6.63. We repeated the process to calculate the CV of mean number of eggs from 10 egg subsamples of the same females and, not surprisingly, values of CV were much higher in this case (range: 10.9–85.9). However, the Gini coefficient for the CVs of mass was actually slightly higher than that for egg number, i.e. 0.268 *vs* 0.236.

In the studies dealing with worm mass in natural populations, the highest inequalities have been recorded for *T. crassus*, with G = 0.61 (overall sample), and 0.59 (average per infrapopulation) [8]. The value is also high for *R. acus* (average G = 0.60 per infrapopulation of gravid females) [10]. The values for *C. cetaceum* are substantially lower (G = 0.314 and G = 0.216 for the overall sample of females and gravid females, respectively), being roughly similar to those reported for females of *Ascaris lumbricoides* in humans (0.25–0.29 for overall samples) [12]. In part, these differences might have to do with profound differences in the lifestyle of each parasite group, most importantly indeterminate *vs* determinate growth. In species with a determinate pattern of growth, such as nematodes or acanthocephalans, the rate of growth is assumed to decrease with age [6]. Accordingly, initial inequalities could be less amplified during subsequent growth, and the level of inequality should decline as the population approaches a stable age/size distribution [6]. This hypothesis could explain why G values significantly decreased from stage-1 to stage-3 females of *C. cetaceum*. It should be noted that there was a linear increase of mass among stages, but this does not imply that growth rate is constant because we have no data on the timing of transition between stages. For instance, based on relative abundance, worms could pass through stage-2 quickly compared to stage-1 and 3 (Additional file 1: Table S1).

Several factors have been invoked to account for body size inequalities in helminths [6, 12]. First, there are genetically-based, individual differences in size and growth rate among worms [6]. Secondly, host individuals can vary in their physiological, immunological or nutritional status, and this variation could influence G values calculated at component community level. Nutritional factors may especially affect the quality of microhabitat conditions because acanthocephalans obtain nutrients directly from host’s diet [15] and, therefore, individual variation in the levels of critical nutrients that each host can provide may contribute to a lower or greater reproductive inequality in their acanthocephalan infrapopulations from host to host [6]. Unfortunately, the reasons for individual variability in both parasites and hosts are rarely known in observational studies and, therefore, we must treat them as random error (parasite individuals) or a random factor (host individuals) in predictive models.

Thirdly, size variability may depend on individual differences in the time since establishment in the host, which in turn depends on the mode of parasite recruitment [12]. In particular, variability will be lower when more worms are simultaneously recruited in each infection event [12, 13]. *Corynosoma cetaceum* infects cetaceans through fish prey, and the diet of franciscanas in the study area includes 8 fish species, of which two, the striped weakfish, *Cyanoscion guatucupa*, and the rough scad, *Trachurus lathami*, make up over 85% of individual prey by number [26]. In samples of these fish species from Buenos Aires Province, both the prevalence (< 35%) and mean intensity (maximum value < 5, with SD per sample < 7 on average) of *C. cetaceum* are low [27, 28]. It is therefore possible that cystacanths of *C. cetaceum* recruit to franciscanas through “trickle” infections [12], a process that would contribute to body size inequalities through variable time of recruitment and growth. We attempted to reduce the effect of time since recruitment by dividing females into three stages; in fact, G was lower for females of each stage than for the overall sample. However, as body growth and egg production are continuous processes, each stage surely contains females of different ages and associated mass. In this context, it is worth noting that (i) based on data from experimental infections, it is likely that the lifespan of *Corynosoma* spp. in their definitive hosts does not exceed 3–4 months [29], and (ii) all franciscanas examined in the austral spring (October-December) of three consecutive years, including those from the present study, were heavily infected with *C. cetaceum* [16]. Together, these observations suggest that the infrapopulations analyzed here were composed of worms continuously recruited over a relatively short period and, therefore, larger infrapopulations likely resulted from more recruitment events. This phenomenon would contribute to generate an overall positive relationship between intensity and inequality, such as we observed.

A fourth factor that has been associated to reproductive inequalities on helminths is intensity-dependence [11], although few studies have actually proven its effects [6, 7]. In theory, intensity-dependence may lead to intraspecific competition between worms and/or an activation of the immune response by the host, and both factors may exacerbate initial inequalities in body size, particularly in helminths with indeterminate growth [6]. Our analysis for *C. cetaceum* reveals a clear effect of intensity on G, but only for stage-2 and stage-3 (i.e. gravid) females. Interestingly, the relationship was conserved after controlling for microhabitat variability, and was stronger when the number of gravid females was used as an alternative predictor of intensity. Thus, there is the possibility that the physiological demands for egg production lead to intraspecific competition between females which, in turn, brings about an intensity-dependent effect on reproductive inequalities (after all, the mass of gravid females indirectly reflects the number and size of eggs they contain). This effect has been shown experimentally in another acanthocephalan species, *M. moniliformis* [6]. At first glance, this hypothesis would not seem consistent with the previous observation that gravid females of *C. cetaceum* do not experience ‘crowding’ effects [16]. However, intraspecific competition could augment individual differences in reproductive success without a net effect upon the average reproductive performance within infrapopulations. In other words, some females would increase in mass at the expense of other females through the differential acquisition of the limited resource. This decoupling between individual (inequality) and population (average) effects has been reported in previous studies (see Background).

Since our system is not amenable to experimental work, it is difficult to shed light on the potential mechanisms whereby intraspecific competition could augment mass inequalities in gravid females of *C. cetaceum*. On the one hand, it is unclear what types of nutrients could be available for an acanthocephalan in the stomach and the upper duodenum, where food is only partly digested [16]. On the other hand, physical and chemical conditions change notably among gut chambers (see Background). However, these microhabitat differences do not appear to affect, on average, the mass and fecundity of *C. cetaceum* [16]. Although we could not directly compare mass inequalities per chamber due to small sample sizes, the SD of the mean worm (the index of microhabitat variability) positively covaried with G for stage-3 females. However, these females also expanded microhabitat use at increasing numbers and, therefore, the microhabitat effect on G could actually be confounded by an overall intensity-dependent effect. In other words, the microhabitat expansion of *C. cetaceum* could (i) have nothing to do with the actual effect of intensity on inequalities or (ii) could generate inequalities assuming that the effects of microhabitat variability show up only at high intensity. The partial correlation analysis lends support to both possibilities since the association between the SD of the mean worm and G dropped when the effect of the number of gravid females was controlled for.

## Conclusions

Females of *C. cetaceum* in franciscanas have modest levels of mass, and presumably fecundity, inequalities. Values are similar to those found in a helminth species with determinate growth that infect mammals, namely *A. lumbricoides*. However, it is unclear whether more general patterns of differences in reproductive inequality exist, e.g. between helminths with determinate *vs* indeterminate growth [6], or from vertebrate *vs* invertebrate hosts [7, 13]. On the other hand, the mass inequalities decrease in gravid *vs* non-gravid females, such as it might be expected in a species with determinate growth. Even so, only gravid females experience intensity-dependent effects which result in higher inequalities of mass *via* individual differences in the access to a limited (trophic) resource and/or a microhabitat expansion that expose worms to a variety of environmental conditions. Although evidence suggests that females of *C. cetaceum* do not differ much in their offspring contribution to the next generations, the present study reveals subtle population effects beyond the common focus on “average” patterns [16].

## Additional files

Additional file 1: **Table S1.** Number of females^*^ of the acanthocephalan *Corynosoma cetaceum* classified into three developmental stages (1–3, see Methods) in three gut chambers from 10 franciscana dolphins, *Pontoporia blainvillei*, which are ordered by increasing intensity. Numbers in parentheses indicate the percentage that each stage represents for the females of each chamber. (DOCX 17 kb)

Additional file 2: **Table S2.** Number of individuals (*n*) and sex ratio (percent males) per gut chamber, and total number of females (F) and males (M), of the acanthocephalan *Corynosoma cetaceum* in 10 franciscana dolphins, *Pontoporia blainvillei*, ordered by increasing intensity. (DOCX 14 kb)

## Abbreviations

DA: Duodenal ampulla
G: Gini coefficient
MS: Main stomach
PS: Pyloric stomach

## References

[1] May RM, Anderson RM (1983). Epidemiology and genetics in the coevolution of parasites and hosts. Proc Royal Soc B..

[2] Gandon S, Day T (2009). Evolutionary epidemiology and the dynamics of adaptation. Evolution..

[3] Papkou A, Gokhale CS, Traulsen A, Schulenburg H (2016). Host-parasite coevolution: why changing population size matters. Zoology..

[4] Anderson RM, May RM (1978). Regulation and stability of host-parasite population interactions, I: Regulatory processes. J Anim Ecol.

[5] Lipsitch M, Nowak MA, Ebert D, May RM (1995). The population dynamics of vertically and horizontally transmitted parasites. Proc Biol Sci..

[6] Dobson AP (1986). Inequalities in the individual reproductive success of parasites. Parasitology..

[7] Hanelt B (2009). An anomaly against a current paradigm: extremely low rates of individual fecundity variability of the Gordian worm (Nematomorpha: Gordiida). Parasitology..

[8] Shostak AW, Dick TA (1987). Individual variability in reproductive success of *Triaenophorus crassus* Forel (Cestoda: Pseudophyllidea), with comments on use of the Lorenz curve and Gini coefficient. Can J Zool..

[9] Beck MA, Goater CP, Colwell DD (2015). Comparative recruitment, morphology and reproduction of a generalist trematode, *Dicrocoelium dendriticum*, in three species of host. Parasitology..

[10] Szalai AJ, Dick TA (1989). Differences in numbers and inequalities in mass and fecundity during the egg-producing period for *Raphidascaris acus* (Nematoda: Anisakidae). Parasitology..

[11] Poulin R, Latham ADM (2002). Inequalities in size and intensity-dependent growth in a mermithid nematode parasitic in beach hoppers. J Helminthol..

[12] Walker M, Hall A, Basánez M-G (2010). Trickle or clumped infection process? An analysis of aggregation in the weights of the parasitic roundworm of humans, *Ascaris lumbricoides*. Int J Parasitol..

[13] Maure F, Poulin R (2016). Inequalities in body size among mermithid nematodes parasitizing earwigs. Parasitol Res..

[14] Keymer A, Crompton DWT, Singhvi A (1983). Mannose and the 'crowding effect' of *Hymenolepis* in rats. Int J Parasitol..

[15] Keymer A, DWT C, Walters DE (1983). Parasite population biology and host nutrition: dietary fructose and *Moniliformis* (Acanthocephala). Parasitology..

[16] Aznar FJ, Bush AO, Balbuena JA, Raga JA (2001). *Corynosoma cetaceum* in the stomach of franciscanas, *Pontoporia blainvillei* (Cetacea): an exceptional case of habitat selection by an acanthocephalan. J Parasitol..

[17] Harrison RJ, Johnson FR, Young BA (1970). The oesophagus and stomach of dolphins (*Tursiops*, *Delphinus*, *Stenella*). J Zool..

[18] Yamasaki F, Takahasi K, Kamiya T (1974). Digestive tract of La Plata dolphin, *Pontoporia blainvillei*. I. Oesophagus and stomach. Okajimas Folia Anat Jpn..

[19] Yamasaki F, Takahasi K, Kamiya T (1975). Digestive tract of La Plata dolphin, *Pontoporia blainvillei*. II. Small and large intestines. Okajimas Folia Anat Jpn..

[20] Bates RM, Kennedy CR (1990). Interactions between the acanthocephalans *Pomporhynchus laevis* and *Acanthocephalus anguillae* in rainbow trout: Testing an exclusion hypothesis. Parasitology.

[21] Weiner J, Solbrig OT (1984). The meaning and measurement of size hierarchies in plant populations. Oecologia..

[22] Moore J, Simberloff D (1990). Gastrointestinal helminth communities of bobwhite quail. Ecology..

[23] Zeileis A. Ineq: Measuring Inequality, Concentration, and Poverty, R package, v0.2-10; 2012. http://cran.r-project.org/web/packages/ineq/ineq.pdf. Accessed 5 Feb 2018.

[24] Handcock MS. Relative distribution methods v1.6-4; 2015. http://CRAN.R-project.org/package=reldist. Accessed 5 Feb 2018.

[25] Canty A, Ripley B. boot: Bootstrap R (S-Plus) Functions, v1.2–42 (R package); 2012. http://cran.r-project.org/package=boot. Accessed 5 Feb 2018.

[26] Paso-Viola MN, Denuncio P, Negri MF, Rodriguez D, Bastida R, Cappozzo HL (2014). Diet cmposition of franciscana dolphin, *Pontoporia blainvillei*, from southern Buenos Aires, Argentina and its interaction with fisheries. Rev Biol Mar Oceanogr..

[27] Timi JT, Luque JL, Sardella NH (2005). Parasites of *Cynoscion guatucupa* along South American Atlantic coasts: evidence for stock discrimination. J Fish Biol..

[28] Braicovich PE, Luque JL, Timi JT (2012). Geographical patterns of parasite infracommunities in the rough scad, *Trachurus lathami* Nichols, in the Southwestern Atlantic Ocean. J Parasitol..

[29] Castro M, Martínez R (2004). Process of the development of *Corynosoma obtuscens* (Acanthocephala: Polymorphidae) in *Canis familiaris* and its possible involvement in public health. Parasitol Latinoam..

