# Supplementary material for: Reproductive inequalities in the acanthocephalan Corynosoma cetaceum: looking beyond ‘crowding’ effects
Source: Parasit Vectors. 2018 Mar 21;11:203. doi: 10.1186/s13071-018-2723-x (PMC5863374; doi:10.1186/s13071-018-2723-x)
Supplement: Supplementary file 1 — Table S1. Number of females* of the acanthocephalan Corynosoma cetaceum classified into three developmental stages (1–3, see Methods) in three gut chambers from 10 franciscana dolphins, Pontoporia blainvillei, which are ordered by increasing intensity. Numbers in parentheses indicate the percentage that each stage represents for the females of each chamber. (DOCX 17 kb) [file 13071_2018_2723_MOESM1_ESM.docx]

**Additional file 1: Table S1.** Number of females^*^ of the acanthocephalan *Corynosoma cetaceum* classified into three developmental stages (1-3, see Methods) in three gut chambers from 10 franciscana dolphins, *Pontoporia blainvillei*, which are ordered by increasing intensity. Numbers in parentheses indicate the percentage that each stage represents for the females of each chamber.

| **Host** | **Main stomach** | | |  | **Pyloric stomach** | | |  | **Duodenal Ampulla** | | |
| --- | --- | --- | --- | --- | --- | --- | --- | --- | --- | --- | --- |
|  | **1** | **2** | **3** |  | **1** | **2** | **3** |  | **1** | **2** | **3** |
| N88-2 | 10  (90.9) | -  - | 1  (9.1) |  | 21  (58.3) | 7  (19.4) | 8  (22.2) |  | 3  (60.0) | 2  (40.0) | -  - |
| N88-4 | 5  (41.7) | 2  (16.7) | 5  (41.7) |  | 6  (19.4) | 8  (25.8) | 17  (54.8) |  | 1  (12.5) | 5  (62.5) | 2  (25.0) |
| N90-17 | 3  (15.0) | 8  (40.0) | 9  (45.0) |  | 11  (15.3) | 17  (23.6) | 44  (61.1) |  | 3  (21.4) | 2  (14.3) | 9  (64.3) |
| N89-19 | 5  (50.0) | 1  (10.0) | 4  (40.0) |  | 39  (24.1) | 28  (17.3) | 95  (58.6) |  | 6  (17.6) | 4  (11.8) | 24  (70.6) |
| N89-2 | 1  (11.1) | -  - | 8  (88.9) |  | 29  (6.9) | 34  (8.0) | 360  (85.1) |  | 4  (5.6) | 1  (1.4) | 66  (93.0) |
| N89-10 | 6  (85.7) | 1  (14.3) | -  - |  | 209  (62.8) | 104  (31.2) | 20  (6.0) |  | 18  (69.2) | 6  (23.1) | 2  (7.7) |
| N89-7 | 9  (69.2) | 2  (15.4) | 2  (15.4) |  | 226  (33.2) | 52  (7.6) | 402  (59.)1 |  | 40  (36.4) | 9  (8.2) | 61  (55.5) |
| N89-14 | 72  (73.5) | 6  (6.1) | 20  (20.4) |  | 218  (23.1) | 107  (11.4) | 617  (65.5) |  | 9  (13.0) | 3  (4.3) | 57  (82.6) |
| N90-18 | 28  (43.8) | 1  (1.6) | 35  (54.7) |  | 181  (22.9) | 53  (6.7) | 555  (70.3) |  | 12  (10.8) | 8  (7.2) | 91  (82.0) |
| N89-17 | 124  (79.5) | 12  (7.7) | 20  (12.8) |  | 287  (36.4) | 147  (18.7) | 354  (44.9) |  | 154  (55.8) | 45  (16.3) | 77  (27.9) |

^*^Some values do not sum up the no. of total females reported in **Table 2**, because some worms were broken and could not be assigned to specific stages.
