# Supplementary material for: Reproductive inequalities in the acanthocephalan Corynosoma cetaceum: looking beyond ‘crowding’ effects
Source: Parasit Vectors. 2018 Mar 21;11:203. doi: 10.1186/s13071-018-2723-x (PMC5863374; doi:10.1186/s13071-018-2723-x)
Supplement: Supplementary file 2 — Table S2. Number of individuals (n) and sex ratio (percent males) per gut chamber, and total number of females (F) and males (M), of the acanthocephalan Corynosoma cetaceum in 10 franciscana dolphins, Pontoporia blainvillei, ordered by increasing intensity. (DOCX 14 kb) [file 13071_2018_2723_MOESM2_ESM.docx]

**Additional file 2: Table S2.** Number of individuals (n) and sex ratio (percent males) per gut chamber, and total number of females (F) and males (M), of the acanthocephalan *Corynosoma cetaceum* in 10 franciscana dolphins, *Pontoporia blainvillei*, ordered by increasing intensity.

| **Host** | **Chamber** | | | | | |  | **Total number** | | |  | **Intensity** |
| --- | --- | --- | --- | --- | --- | --- | --- | --- | --- | --- | --- | --- |
|  | **Main stomach** | | **Pyloric stomach** | | **Duodenal ampulla** | |  | **F** | **M** | **Sex ratio** |  |  |
|  | **n** | **Sex ratio** | **n** | **Sex ratio** | **n** | **Sex ratio** |  |  |  |  |  |  |
| N88-2 | 28 | 57.1 | 61 | 39.3 | 10 | 50.0 |  | 54 | 45 | 45.5 |  | 99 |
| N88-4 | 33 | 63.6 | 68 | 55.9 | 27 | 70.4 |  | 50 | 78 | 60.9 |  | 128 |
| N90-17 | 41 | 46.3 | 163 | 49.1 | 28 | 39.3 |  | 122 | 110 | 47.4 |  | 232 |
| N89-19 | 26 | 61.5 | 346 | 48.6 | 46 | 39.1 |  | 216 | 202 | 48.3 |  | 418 |
| N89-2 | 17 | 47.1 | 594 | 24.7 | 108 | 26.9 |  | 535 | 184 | 25.6 |  | 719 |
| N89-10 | 13 | 46.2 | 685 | 49.9 | 50 | 48.0 |  | 376 | 372 | 49.7 |  | 748 |
| N89-7 | 22 | 40.9 | 1187 | 34.4 | 199 | 43.2 |  | 905 | 503 | 35.7 |  | 1408 |
| N89-14 | 176 | 44.3 | 1549 | 36.9 | 120 | 41.7 |  | 1146 | 699 | 37.9 |  | 1845 |
| N90-18 | 122 | 56.6 | 1481 | 40.6 | 246 | 51.2 |  | 1052 | 797 | 43.1 |  | 1849 |
| N89-17 | 473 | 50.1 | 1595 | 43.3 | 624 | 43.3 |  | 1495 | 1197 | 44.5 |  | 2692 |
